# A Multicenter Randomized Double-Blind Vehicle-Controlled Parallel Group Phase 2 Study Evaluating the Efficacy and Safety of GN-037 Cream in Patients with Mild-to-Moderate Plaque Psoriasis

**DOI:** 10.1007/s13555-024-01301-1

**Published:** 2024-11-22

**Authors:** Burhan Engin, Müge Güler Özden, Özge Sevil Karstarlı Bakay, Selda Pelin Kartal, İlkin Zindancı, Salih Levent Çınar, Recep Dursun, Gizem Pehlivan Ulutaş, Tuğba Özkök Özkök Akbulut, Fatma Aslı Hapa, Emel Bülbül Başkan, Mehmet Melikoğlu, Algün Polat Ekinci, Neslihan Demirel Öğüt, Pelin Hızlı, Zafer Türkoğlu, Özlem Su Küçük, Zeynep Topkarcı, Ümit Türsen, Filiz Canpolat, Hanife Uçgun, Şirin Yaşar, Selami Aykut Temiz, Asena Çiğdem Doğramacı, Sedat Altuğ, Serhat Kozlu, Nadir Ulu, Server Serdaroğlu

**Affiliations:** 1 Cerrahpaşa Faculty of Medicine, İstanbul University-Cerrahpaşa, Istanbul, Türkiye; 2 Faculty of Medicine, Ondokuz Mayıs University, Samsun, Türkiye; 3 Faculty of Medicine, Pamukkale University, Denizli, Türkiye; 4 Etlik City Hospital, Ankara, Türkiye; 5 Haydarpaşa Numune Training and Research Hospital, University of Health Sciences, Istanbul, Türkiye; 6 Faculty of Medicine, Erciyes University, Kayseri, Türkiye; 7 Meram Faculty of Medicine, Necmettin Erbakan University, Konya, Türkiye; 8 Haseki Training and Research Hospital, Istanbul, Türkiye; 9 Buca Seyfi Demirsoy Training and Research Hospital, Democracy University, İzmir, Türkiye; 10 Faculty of Medicine, Uludağ University, Bursa, Türkiye; 11 Faculty of Medicine, Atatürk University, Erzurum, Türkiye; 12 İstanbul Faculty of Medicine, İstanbul University, Istanbul, Türkiye; 13 Uşak Training and Research Hospital, Uşak, Türkiye; 14 Faculty of Medicine, Balıkesir University, Balıkesir, Türkiye; 15 Başakşehir Çam ve Sakura City Hospital, Istanbul, Türkiye; 16 Faculty of Medicine, Bezmialem Vakıf University, Istanbul, Türkiye; 17 Bakırköy Dr Sadi Konuk Training and Research Hospital, University of Health Sciences, Istanbul, Türkiye; 18 Faculty of Medicine, Mersin University, Mersin, Türkiye; 19 Faculty of Medicine, Hatay Mustafa Kemal University, Hatay, Türkiye; 20 Faculty of Medicine, Demiroğlu Bilim University, Istanbul, Türkiye; 21 Department of Research and Development, Gen İlaç ve Sağlık Ürünleri A.Ş., ASO 2. Ve 3. Organize Sanayi Bolgesi, Alci OSB Mahallesi, 2013. Cadde, No: 24, 06930 Sincan, Ankara Türkiye

**Keywords:** GN-037, Topical treatment, Mild-to-moderate plaque psoriasis, Phase 2

## Abstract

**Introduction:**

Topical therapies are used in almost all patients with psoriasis. A novel fixed topical combination cream (GN-037) with a lower concentration (0.0356%) of clobetasol 17-propionate (CP) was developed together with urea, salicylic acid, and retinoic acid to provide a better benefit–risk ratio. The present multicenter randomized double-blind vehicle-controlled parallel group phase 2 study aimed to investigate the efficacy and safety of GN-037 in patients with mild-to-moderate plaque psoriasis (MMPP).

**Methods:**

Patients (*n* = 190) were randomized (2:2:1) to receive GN-037 or CP or vehicle (V) cream twice daily to a selected target body lesion for 4 weeks. The primary endpoint was treatment success defined as percentage of patients with at least two-grade improvement in Investigator’s Global Assessment Score (IGA) and IGA score equal to 0 or 1 evaluated at weeks 2, 4, 6, and 8 in each arm compared with baseline. Treatment-emergent adverse events (TEAEs) and safety were evaluated throughout the study.

**Results:**

GN-037 demonstrated statistically significant superiority over V throughout the study. At week 4, treatment success was achieved in 37.9% of patients in the GN-037 arm compared with 29.2% and 9.1% in the CP and V arms, respectively. At least two-grade improvement compared with baseline was achieved by 57.6%, 72.7%, and 80.3% of the patients in the GN-037 arm for erythema, plaque elevation, and scaling, respectively. The mean changes in affected BSA were −2.1 ± 2.9, −1.8 ± 2.4, and −0.5 ± 1.6 in the GN-037, CP, and V arms, respectively. The TEAEs were similar among the arms and the most frequently observed TEAEs were Psoriasis Area and Severity Index (PASI) increase in all arms.

**Conclusions:**

GN-037 was more effective than V in achieving primary and all secondary endpoints throughout the study. Safety data did not reveal any new safety concerns with the combination cream product. Therefore, 4 weeks of GN-037 treatment demonstrated an excellent efficacy and safety profile in patients with MMPP.

**Trial Registration number:**

ClinicalTrials.gov identifier, NCT05706870.

## Key Summary Points

**Table:** 

Topical combination treatment options that reduce the complexity of treatment can improve patient adherence in plaque psoriasis.
A new topical fixed combination, GN-037 cream, was developed by combining a lower clobetasol 17-propionate (CP) concentration with urea, salicylic acid, and retinoic acid.
GN-037 demonstrated statistically significant superiority over vehicle (V) in patients with mild-to-moderate plaque psoriasis (MMPP) throughout the study.
Safety data did not reveal any new safety concerns with the combination cream product.
GN-037 is an effective and safe new treatment option for patients with MMPP.

## Introduction

Psoriasis is a common multisystemic chronic inflammatory skin disorder that affects large populations, regardless of age. The prevalence rate of psoriasis is approximately 1.5–2.0% worldwide and varies according to geographic regions, is more frequent in countries closer to the equator, and can reach 8.5% in Nordic countries [1]. Although the causes of psoriasis have not yet been fully elucidated, several risk factors have been recognized, including genetic factors, stress, obesity, smoking, and alcohol consumption [2]. Unfortunately, the clinical course of psoriasis is unpredictable, and the main symptoms usually include flaky and scaly skin, itching, swelling, bleeding, and skin damage [3]. Psoriasis vulgaris is the most common subtype of psoriasis and comprises 90% of cases.

Treatment modalities can be classified into three major groups: topical agents, systemic therapies that target specific inflammatory pathways, and phototherapy. The choice of treatment depends on the severity of the disease, comorbidities, and preferences of the patient. Although several new biological therapies are currently available for the treatment of psoriasis, there is still an unmet medical need for topical agents in many patients, as none of the current systemic treatments has been shown to be curative. Moreover, visible plaques and the chronic nature of the disease usually affect the quality of life and psychology of patients, eventually leading to anxiety and depression [4]. A personalized approach that focuses particularly on the severity of the disease, responses to previous treatment, and patient preferences is generally considered for the management of these patients. Maintenance treatment with topical agents, including corticosteroids and vitamin D analogues, has been well accepted as a first-line treatment option in more than two-thirds of patients with MMPP. Since corticosteroids have antiproliferative, antiinflammatory, and local vasoconstrictive properties through the downregulation of proinflammatory cytokine-encoding genes, topical corticosteroids are the standard primary topical treatment for most patients with localized or mild lesions. The duration of remission and/or the frequency of relapses can vary due to patient adherence. However, it is well known that adherence to topical corticosteroid treatment is limited in the real world and is much lower than reported in randomized control trials. For example, adherence to topical therapies in psoriasis can be as low as 61% and even lower with long-term use [5, 6]. Poor adherence can be explained in part by adverse events, dissatisfaction with treatment success, inconvenience of application, cost, and complexity of the regimens [7, 8].

Combination of topical treatment options that reduce the complexity of treatment can improve adherence. As previously reported, long-term topical use of super-high- and high-potent corticosteroids can cause potential side effects, including local skin alterations such as cutaneous atrophy, striae, and systemic side effects such as suppression of the hypothalamic–pituitary–adrenal (HPA) axis, pustular flares, and tachyphylaxis [9]. Therefore, novel formulations containing lower concentrations of commonly prescribed corticosteroids such as CP can provide a better benefit–risk ratio. To this end, a new topical fixed combination, GN-037 cream, was developed by combining a lower concentration of CP concentration with urea, salicylic acid, and retinoic acid.

We have recently demonstrated the safety of GN-037 cream treatment in healthy volunteers and patients with plaque psoriasis. Furthermore, clinically meaningful efficacy was observed in six patients in the phase 1 setting [10]. Therefore, the present phase 2 study aimed to investigate the safety and efficacy of GN-037 in patients with MMPP.

## Methods

### Study Design

This study was a randomized, double-blind, multi-center, vehicle-controlled, parallel group phase 2 study comparing the efficacy, safety, and tolerability of topically applied GN-037 cream against V in the treatment of MMPP (with an IGA score of 2 or 3 and an affected BSA of 3–12%; Table 1). Areas of the face, scalp, palms, soles, axillae, or other intertriginous areas were not included in IGA and BSA calculations.

**Table 1:** Summary of baseline characteristics (ITT population)

	GN-037 (*N* = 76)	CP (*N* = 77)	V (*N* = 37)
Age (years)			
Mean ± SD	40 ± 12.4	40 ± 11.4	41 ± 12.5
Median (min–max)	40 (20–63)	39 (20–64)	41 (18–64)
Sex, *n* (%)			
Female	36 (47.4)	31 (40.3)	17 (45.9)
Male	40 (52.6)	46 (59.7)	20 (54.1)
BMI			
Mean ± SD	28.0 ± 5.3	28.7 ± 5.6	29.3 ± 4.9
Median (min–max)	27.4 (18.0–45.8)	28.0 (19.1–50.5)	28.4 (20.8–39.1)
IGA, *n* (%)			
2-Mild	36 (47.4)	33 (42.9)	18 (48.6)
3-Moderate	40 (52.6)	44 (57.1)	19 (51.4)
%BSA affected by psoriasis			
Mean ± SD	5.7 ± 3	6.4 ± 3.2	6.4 ± 3.2
Median (min–max)	5 (3–12)	5 (3–12)	6 (3–12)
Size of target lesion (cm^2^)			
Mean ± SD	29.0 ± 20.1	29.4 ± 18.8	28.0 ± 18.4
Median (min–max)	20 (16–100)	20 (16–100)	20 (16–96)

*ITT* intent-to-treat, *GN-037* investigational novel fixed topical combination, *CP* clobetasol 17-propionate, *V* vehicle, *BMI* body mass index, *IGA* Investigator’s Global Assessment Score, *BSA* body surface area

Patients (*n* = 190) were randomized (2:2:1 ratio) to receive GN-037 or CP or V twice daily to a selected target body lesion for 4 weeks. The total duration of the study was 8 weeks consisting of six visits: screening, baseline (day 1), and weeks 2, 4, 6, and 8. Remote online visits were also conducted (weeks 1, 3, 5, and 7) to monitor adverse events (AEs). The patients were enrolled in 19 centers in Türkiye between December 2022 and January 2024.

The Ethics Committee for Clinical Trials of Istanbul University-Cerrahpasa, Cerrahpasa Faculty of Medicine, Istanbul, Türkiye (approval date: 04/10/2022) and the Turkish Medicines and Medical Devices Agency approved the study protocol (approval date: 03/11/2022). The trial was registered at www.clinicaltrials.gov (NCT05706870) and was carried out according to the principles of the Declaration of Helsinki and the current version of ICH-GCP (E6) and local regulatory guidelines. Written informed consent was obtained from all patients prior to enrollment.

### Eligibility Criteria

Female and male patients with MMPP aged 18–65 years were enrolled. Patients diagnosed with MMPP by a dermatologist at least 6 months prior to the study should have received their last psoriasis treatment 4 weeks or earlier before enrollment to be eligible for the study.

Patients with spontaneously resolving or rapidly deteriorating psoriasis (e.g., guttate, erythrodermic, exfoliative, or pustular) that could affect the evaluation of psoriasis were not included in the study. Furthermore, patients with other inflammatory skin diseases in the target treatment areas that could interfere with study assessments (e.g., atopic dermatitis, contact dermatitis, eczema, tinea corporis), having a history of phototherapy, photochemotherapy, systemic or local psoriasis treatments, or systemic antiinflammatory agent use in the last 4 weeks, biological therapy use for psoriasis in the last 3 months, having abnormalities of the HPA axis, having treatment history with dehydroepiandrosterone (DHEA) or dehydroepiandrosterone sulfate (DHEAS) in the last month, being hypersensitive to any component of the investigational product, and having positive pregnancy test were excluded. Furthermore, patients who were pregnant, breastfeeding, corticosteroid resistant/unresponsive, immunocompromised, and hypertensive (severe) or patients receiving immunosuppressive therapy (including those who had received immunosuppressive drugs in the last 2 months) or with a history of cancer treatment in the last year were also excluded from the study.

### Study Treatments

The GN-037 cream contains fixed doses of CP (0.0356%), urea (9.48%), salicylic acid (4.74%), and retinoic acid (0.0012%) as active pharmaceutical ingredients (API). As a control, V was manufactured with the same appearance, color, and odor as GN-037 cream, except for API. CP (%0.0356) was used as a comparator, particularly for safety, administered topically in 50-g tubes, similar to GN-037 and V cream.

The patients were instructed how to apply the study treatments topically to cover the target lesion. A thin layer of study treatments was applied twice daily, in the morning and evening, according to the treatment arm for a duration of 4 weeks. At the end of the study, both used and unused tubes were collected, weighed, and recorded for each patient.

### Assessments

Efficacy was evaluated using the IGA, BSA, PASI, and Target Plaque Severity Score (TPSS) at weeks 2, 4, 6, and 8 compared with baseline. The efficacy outcomes were evaluated by examining the target lesion (16 and 100 cm^2^ in size) with a score of ≥ 3 on two or more clinical symptom scales (including erythema, plaque thickness, and scaling) and a total score of ≥ 8, with no scale scoring < 2. The erythema, plaque thickness, and scaling scores of the target lesion were monitored at each onsite visit.

Suppression of the HPA axis was indirectly assessed by monitoring plasma DHEAS levels [by electrochemiluminescence immunoassay (ECLIA)] at weeks 2 and 4 and compared with baseline.

The primary efficacy endpoint was treatment success defined as the percentage of patients with at least a two-grade improvement in the IGA score and achieving an IGA score of 0 or 1 assessed at weeks 2, 4, 6, and 8 in each arm compared with baseline. Other endpoints included improvement in the PASI-75 score, mean changes in BSA and TPSS of the target lesion compared with baseline, and the number and percentage of patients who improved at least two points in erythema, plaque thickness, and scaling scores of the target lesion at weeks 2, 4, 6, and 8 compared with baseline.

For safety assessments, adverse events (AE), serious adverse events (SAE), physical examination, vital signs and laboratory parameters were recorded and monitored at each visit. The tolerability of the local treatment area was evaluated on the basis of symptoms such as itching, local burning, pain, skin atrophy, striae, and telangiectasia, and the severity of any reactions was recorded. Improvement in the Dermatology Life Quality Index (DLQI) was also assessed at baseline and weeks 4 and 8.

### Data Analysis

The primary objective of this study was to evaluate the efficacy of the GN-037 investigational product. The secondary objective was to determine the safety of GN-037 in patients with MMPP compared with CP and V.

The efficacy analyses were performed on both the per-protocol (PP) and the intent-to-treat (ITT) population. The safety population included all randomized patients who had received at least one dose of the study drug.

The sample sizes of 76 patients in each treatment arm and 38 patients in the V arm achieved 98% power to detect a difference of 0.3320 between the group proportions. Under the null hypothesis, the proportion in the treatment arm was assumed to be 0.0970, and under the alternative hypothesis, this proportion was assumed to be 0.4290. The proportion in the V arm was 0.0970. This statistic was calculated using the Mantel–Haenszel test, with a significance level of 0.05 as the target. The actual significance level obtained with this design was calculated as 0.0423.

The demographic and clinical findings of the patients were evaluated according to the treatment arms. Descriptive statistics such as mean, standard deviation, or median (minimum–maximum) for continuous variables, and frequencies (*n*) and percentages for categorical variables, were provided for the arms. The chi-squared test (or Fisher Exact/Yates continuity correction) was used for the comparison of categorical variables, while continuous variables were compared by using the analysis of variance (ANOVA) test (for normally distributed variables) or the Kruskal–Wallis test (for nonnormally distributed data). Post hoc comparisons were performed for statistically significant results. To maintain the desired level of type I error, the Bonferroni correction was used. Changes in clinical scores for each treatment arm were evaluated using the paired sample *t*-test or the Wilcoxon test. Statistical significance was established at 0.05. All statistical analyses were performed with SPSS (IBM SPSS Statistics for Macos, Version 26.0. Armonk, NY). Statistical differences between treatment arms were analyzed using Graph Pad Prism 9 statistical software (Boston, MA).

The incidence of AEs was based on the number of subjects in the respective analysis population and treatment arm. AEs were coded according to the Medical Dictionary for Regulatory Activities (MedDRA) for reporting at the ‘‘Preferred Terms’’ level.

## Results

### Subject Demographics

**Fig. 1:**
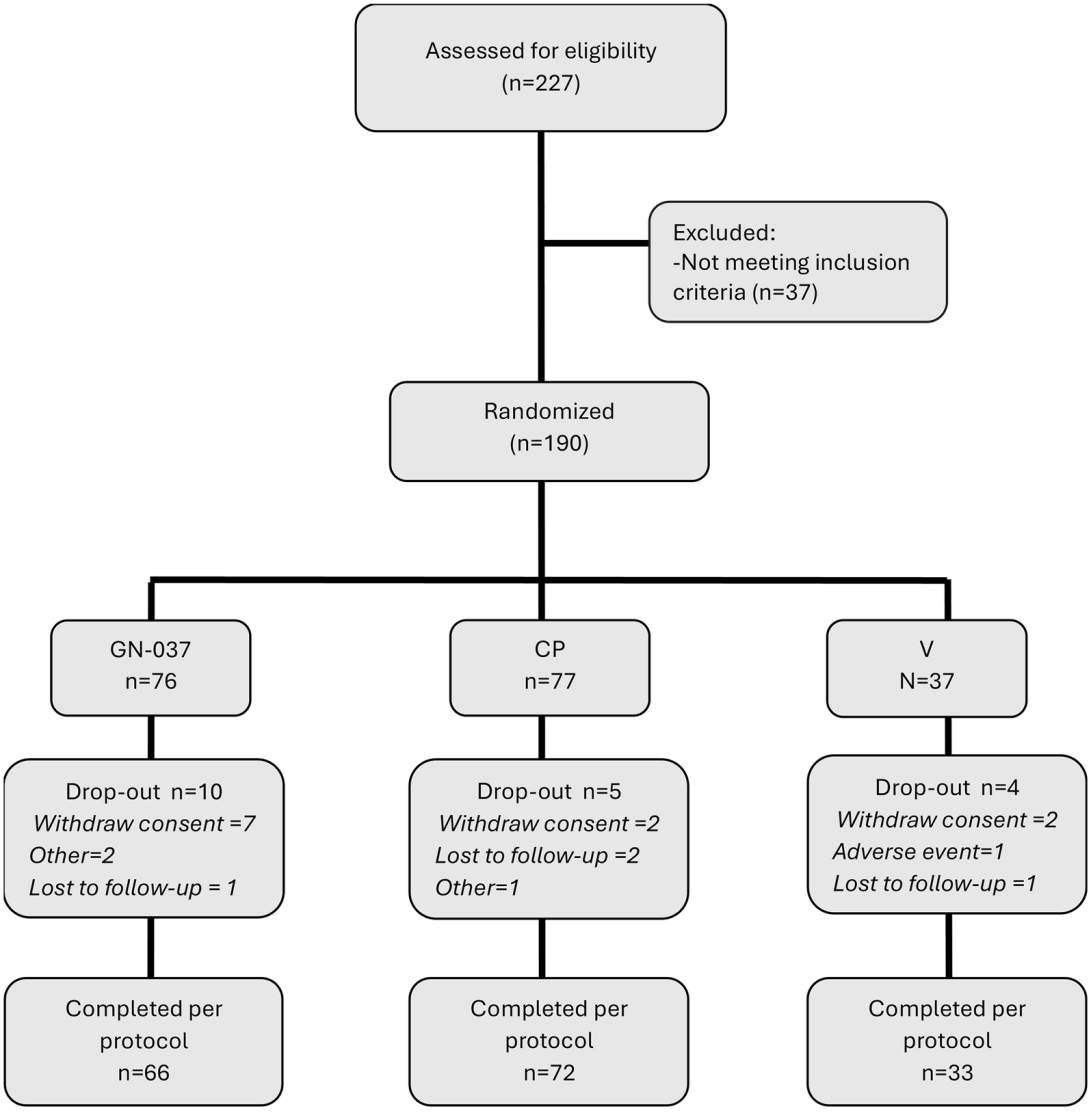
Patient recruitment and randomization flow chart

A total of 227 participants were screened between 28 December 2022 and 4 January 2024, and 37 out of 227 were evaluated as ineligible during the screening process (Fig. 1). A total of 190 participants [106 men (55.8%) and 84 women (44.2%)] who met the inclusion/exclusion criteria were randomized to the GN-037 (*n* = 76), CP (*n* = 77), and V (*n* = 37) arms. Overall, 171 patients completed the study according to the protocol; 66 (86.8%) in the GN-037, 72 (93.5%) in the CP, and 33 (89.2%) in the V arms.

A total of 19 patients did not complete the study (Fig. 1). Among them, 11 patients withdrew their informed consents and 4 patients were lost to follow-up. Only one patient in the V arm discontinued due to an adverse event. It is important to emphasize that Türkiye experienced two subsequent devastating earthquakes with a magnitude of 7.8 Mw and 7.5 Mw, respectively, in the southeast part of the country on 6 February 2023. The clinical center in the city of Hatay had to be closed and two randomized patients from this center in the GN-037 arm discontinued due to the earthquake.

There were no statistically significant differences in demographic variables between the arms (Table 1). The median age was 40, 39, and 41 years and the mean body mass index was 28.0, 28.7, and 29.3 kg/m^2^, in the GN-037, CP, and V arms, respectively (Table 1).

### Efficacy Assessment

Efficacy assessments were analyzed both in the ITT and PP populations at weeks 2, 4, 6, and 8 compared with baseline. GN-037 demonstrated statistically significant superiority over V throughout the study.

**Fig. 2:**
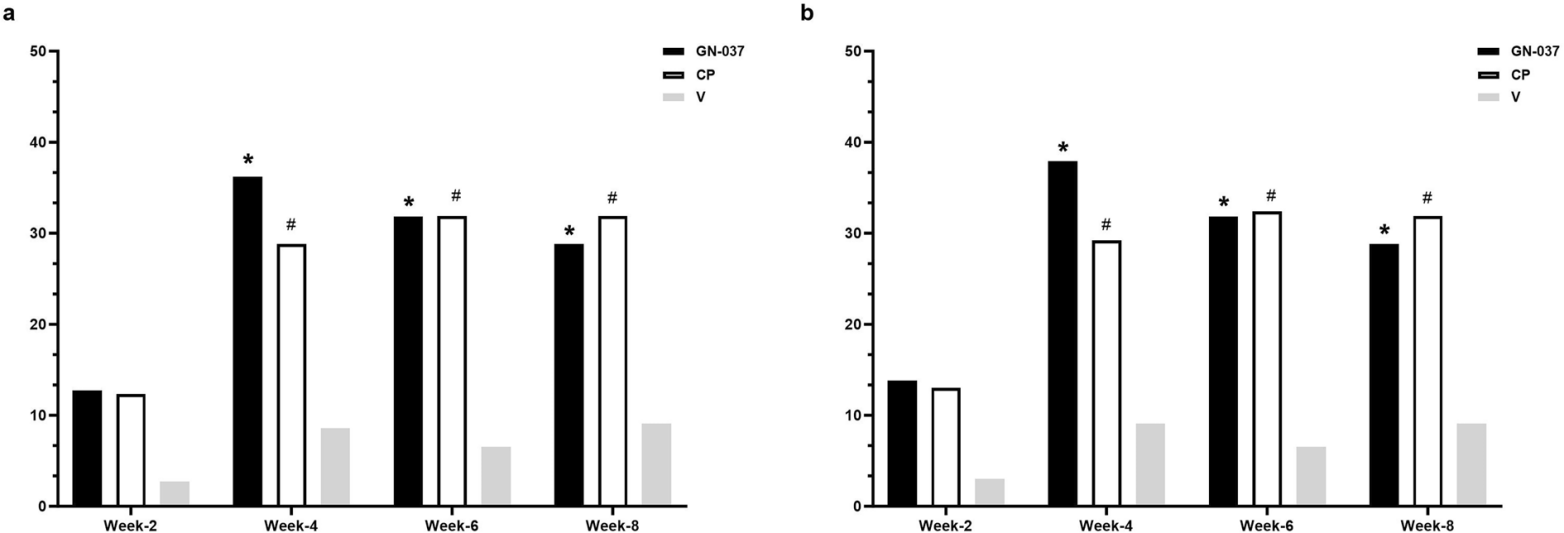
Treatment success throughout the study period. Treatment success is defined as an improvement of at least 2 points in the IGA score from baseline, resulting in a final score of 0 or 1. **a.** ITT population and **b.** PP population. *P < 0.05, GN-037 versus V, ^#^*P* < 0.05, CP versus V

**Fig. 3:**
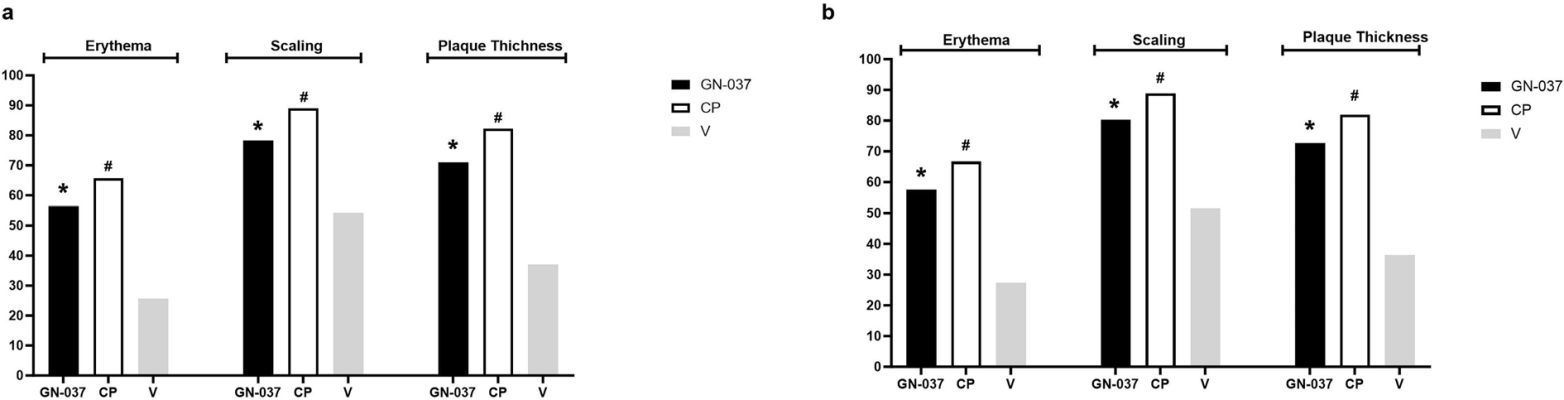
Improvement in reducing psoriasis signs of erythema, scaling, and plaque elevation at week 4. The figure demonstrates the percentage of patients who achieved at least a 2-point improvement in erythema, scaling, and plaque elevation scores compared with baseline. **a.** ITT population and **b.** PP population. *P < 0.05, GN-037 versus V, ^#^*P* < 0.05, CP versus V

**Fig. 4:**
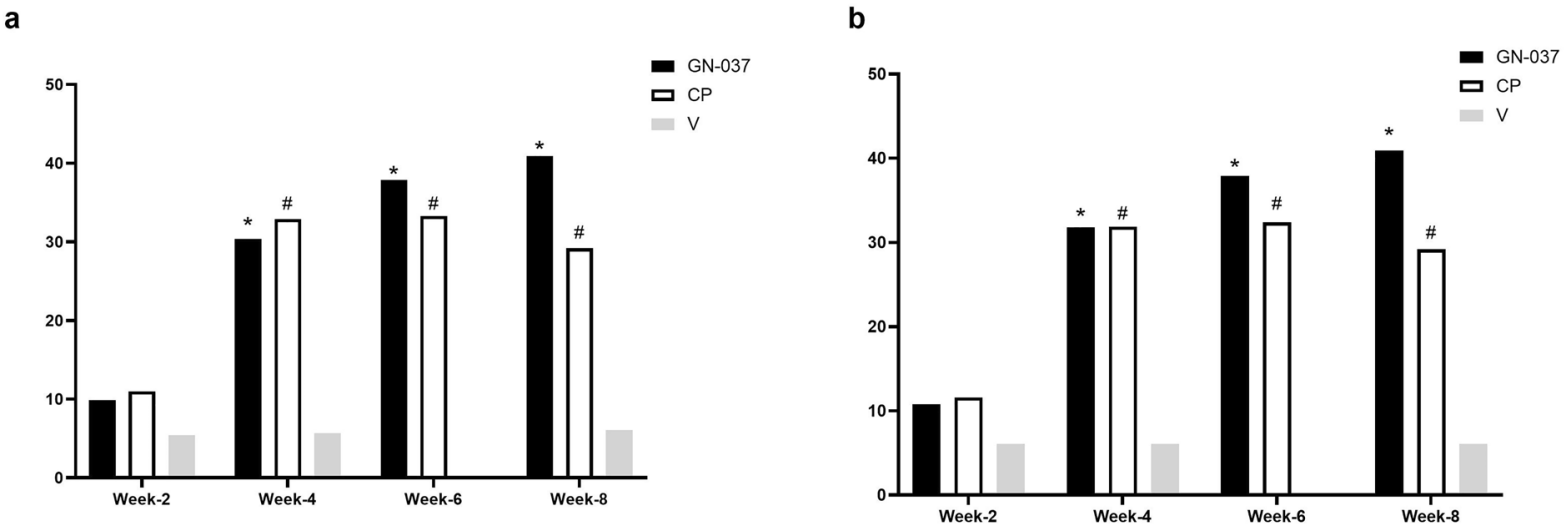
Improvement in PASI-75. The figure demonstrates the improvement in the PASI-75, showing the percentage of patients who achieved at least a 75% reduction in PASI score compared with baseline. **a.** ITT population and **b.** PP population. *P < 0.05, GN-037 versus V, ^#^*P* < 0.05, CP versus V

**Fig. 5:**
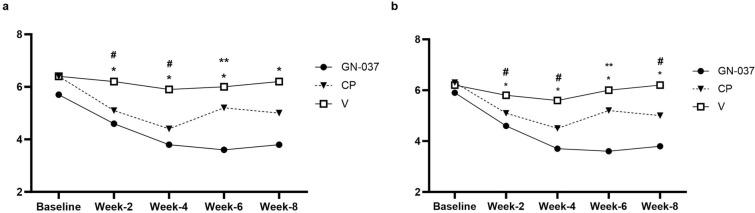
The mean changes in affected BSA. The figure demonstrates the mean changes in the affected BSA throughout the study period. **a.** ITT population and **b.** PP population. *P < 0.05, GN-037 versus V, ^#^*P* < 0.05, CP versus V, **P < 0.05 GN-37 versus CP

**Fig. 6:**
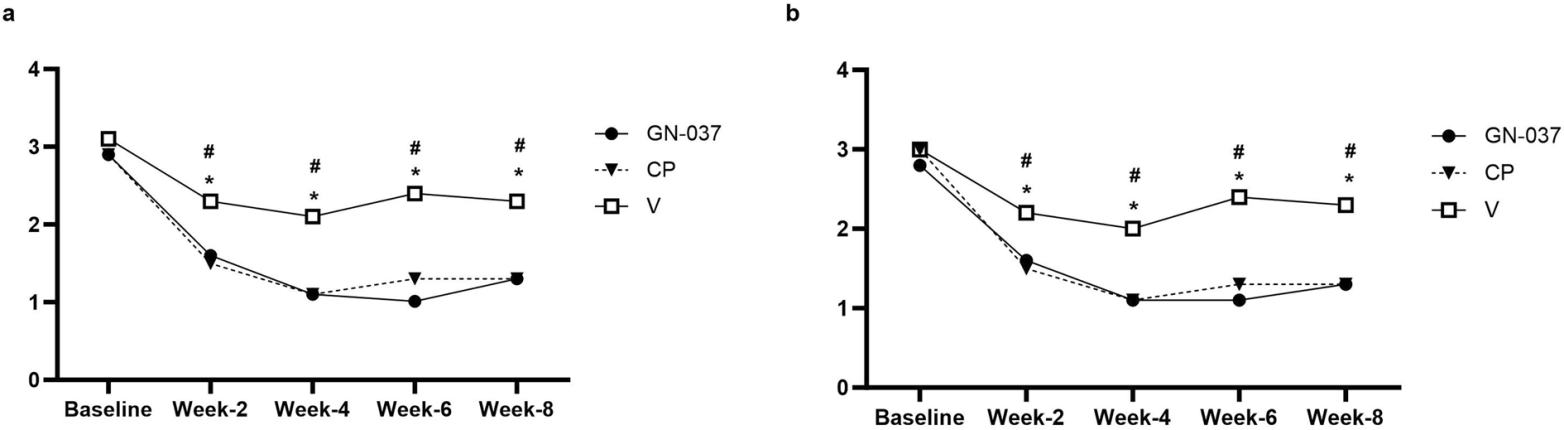
The mean changes in TPSS. The figure demonstrates the mean changes in TPSS throughout the study period. **a.** ITT population and **b.** PP population. *P < 0.05, GN-037 versus V, ^#^*P* < 0.05, CP versus V

In the ITT population at week 4, treatment success was achieved in 36.2% of the patients in the GN-037 arm compared with 28.8% in the CP (*P* = 0.441) and 8.6% in the V (*P* = 0.006) arms (Fig. 2a). GN-037 was also superior to V in reducing the signs of erythema, plaque elevation, and scaling. At least two-grade improvements compared with baseline were achieved at week 4 by 56.5% (*P* < 0.001 versus V), 71.0% (*P* < 0.001 versus V), and 78.3% (*P* < 0.001 versus V) of patients in the GN-037 arm for erythema, plaque elevation, and scaling, respectively (Fig. 3a). At week 4, improvement in PASI-75 was achieved in 30.4% (*P* = 0.009 versus V), 32.9% (*P* = 0.004 versus V), and 5.7% of patients (Fig. 4a) and mean changes in affected BSA were −2.1 ± 2.9 (*P* = 0.006 versus V), −1.8 ± 2.4 (*P* = 0.038 versus V) and −0.5 ± 1.6 (Fig. 5a) in the GN-037, CP, and V arms, respectively. The mean changes in the affected BSA at week 6 were −2.2 ± 2.9 (*P* = 0.002 versus V and *P* = 0.029 versus CP), −1.00 ± 3.01 (*P* = 0.451 versus V), and −0.15 ± 1.81 (Fig. 5a) in the GN-037, CP, and V arms, respectively (Fig. 5a). The mean changes in TPSS at week 4 were −1.70 ± 1.06 (*P* = 0.002 versus V), −1.85 ± 1.14 (*P* < 0.001 versus V), and −0.91 ± 1.04 (Fig. 6a) in the GN-037, CP, and V arms, respectively.

We also measured DLQI scores at baseline and weeks 4 and 8. In the PP population, baseline median DLQI scores were 9.5, 10, and 10 in the GN-037, CP, and V arms, respectively. Treatment with GN-037 for 4 weeks resulted in a trend toward a reduction in median DLQI scores that was maintained until week 8 (Table 2).

**Table 2:** Median (min–max) dermatology life quality index (DLQI) scores at baseline and weeks 4 and 8 (PP population)

	GN-037 (*n* = 66)	CP (*n* = 72)	V (*n* = 33)
Baseline	9.5 (0–23)	10 (0–24)	10 (1–30)
Week 4	7 (0–24)	9 (1–30)	10 (1–26)
Week 8	7 (0–30)	9.5 (0–26)	10 (0–28)

*PP* per-protocol, *GN-037* investigational novel fixed topical combination, *CP* clobetasol 17-propionate, *V* vehicle

### Plasma CP Concentrations

Plasma CP was detectable only in five patients (7.6%) in GN-037 and in two patients (2.8%) in CP arms. In these patients mean plasma CP concentrations were 129.98 ± 35.13 and 124.71 ± 32.37 pg/mL in the GN-037 and CP arms, respectively.

### Safety

Safety assessments were carried out in the safety population. A total of 420 adverse events (146 in the GN-037, 167 in the CP, and 107 in the V arms) were reported by 190 randomized patients who received at least one dose of the study drug. Almost all TEAEs were considered mild in severity, and only two cases of increase in PASI score were reported to be moderate in the V arm. There were no significant differences in laboratory values, vital signs, electrocardiograms, or physical (non-dermatological) examinations between the arms. There were no SAEs or deaths.

The TEAEs were similar among the arms and the most frequently observed TEAEs were increased PASI in all arms (Table 3, 5.5% in GN-037, 7.2% in CP, and 7.5% in V arms). Skin reactions, such as pruritus, dry skin, rash, and postinflammatory pigmentation change, were rare in all arms (3.42% in the GN-037, 3.59% in the CP, and 5.61% in the V arms, respectively), and no skin atrophy or skin infection was observed.

**Table 3:** Treatment-emergent adverse events in the safety population

	GN-037	CP	V	
	(*n* = 146)	(*n* = 167)	(*n* = 107)	
	Mild	Mild	Mild	Moderate
	*n* = 15	*n* = 18	*n* = 8	*n* = 2
	*n* (%)	*n* (%)	*n* (%)	*n* (%)
Investigations	9 (6.2%)	12 (7.2%)	6 (5.6%)	2 (1.9%)
Psoriasis area severity index increase	8 (5.5%)	12 (7.2%)	6 (5.6%)	2 (1.9%)
Dehydroepiandrosterone increase	1 (0.7%)			
General disorders and administration site conditions	5 (3.4%)	3 (1.8%)		
Application site burn	2 (1.4%)	3 (1.8%)		
Application site exfoliation	1 (0.7%)			
Application site hypertrichosis	1 (0.7%)			
Application site erythema	1 (0.7%)			
Skin and subcutaneous tissue disorders	1 (0.7%)	2 (1.2%)	1 (0.9%)	
Postinflammatory pigmentation change	1 (0.7%)	1 (0.6%)		
Pruritus			1 (0.9%)	
Dry skin		1 (0.6%)		
Endocrine disorders		1 (0.6%)	1 (0.9%)	
Adrenocorticotropic hormone deficiency		1 (0.6%)	1 (0.9%)	

*GN-037* investigational novel fixed topical combination, *CP* clobetasol 17-propionate, *V* vehicle

### DHEAS Levels

The baseline mean plasma ACTH results were similar in all arms (GN-037, 16.7 pg/mL; CP, 16.8 pg/mL; and V, 20.9 pg/mL). As an indirect measure of HPA axis suppression, plasma DHEAS levels were measured at baseline and weeks 2 and 4. In general, we did not observe any differences in plasma DHEAS levels between arms at all timepoints (Table 4). The mean plasma DHEAS levels in the GN-037 arm were 198.0 ± 110.2 µg/dL at baseline and 187.6 ± 113.2 and 190.0 ± 115.3 µg/dL at weeks 2 and 4, respectively. Similarly, there were no changes in DHEAS levels in the CP and V arms at weeks 2 and 4.

**Table 4:** Mean ± SD dehydroepiandrosterone sulfate (DHEAS) levels (µg/dL) at baseline, weeks 2 and 4 (PP population)

	GN-037 (*n* = 66)	CP (*n* = 72)	V (*n* = 33)
Baseline	198.0 ± 110.2	208.6 ± 115.5	211.6 ± 98.4
Week 2	187.6 ± 113.2	209.8 ± 129.5	213.6 ± 108.5
Week 4	190.0 ± 115.3	204.3 ± 118.9	213.5 ± 111.4

*GN-037* investigational novel fixed topical combination, *CP* clobetasol 17-propionate, *V* vehicle

## Discussion

The results of this phase 2 study in patients with MMPP demonstrate a clear efficacy and a good safety profile of the new fixed combination cream GN-037 that contains CP (0.0356%), urea (9.48%), salicylic acid (4.74%), and retinoic acid (0.0012%). The combination of corticosteroids with other APIs aiming for different activities, such as desquamation of lesions, moistening, and inhibition of skin decolorization, can improve the success of treatment in patients with MMPP. Furthermore, lowering the concentration of corticosteroids without compromising efficacy is one of the ways to overcome potential adverse events related to corticosteroids. Salicylic acid has been used since the 1970s in many compounded topical preparations (CTP) that are commonly used for the treatment of psoriasis [11, 12]. In addition to salicylic acid, retinoic acid that affects keratinocyte functions and immune response is also frequently preferred [13]. It is well established that urea exerts its beneficial effects by improving the barrier function of the skin and increasing the penetration of topical therapies. Therefore, urea-containing formulations or CTPs have been widely used in most dermatosis, including psoriasis, atopic dermatitis, ichthyosis, xerosis, and seborrheic dermatitis [14]. Although different combinations of CTPs of the drugs mentioned above are commonly prescribed as personalized treatment, they are generally prepared by pharmacists, which has some disadvantages, such as the need for specialized equipment and the lack of enough information on the stability, composition, and incompatibilities with APIs. Therefore, GN-037 was developed as a new fixed combination cream.

Results of this phase 2 study demonstrated a comparable efficacy profile to previously published studies with CP cream [15, 16]. A sustained treatment benefit was still evident at week 8 in the GN-037 and CP arms, as evidenced by significantly higher percentages of patients with treatment success in both arms compared with V. This finding was further supported by improvements in PASI-75 and decreases in mean BSA at week 8 in both arms. Remarkably, the positive effect of CP cream on BSA partially disappeared at weeks 6 and 8 after the end of treatment at week 4, while GN-037 continued to be effective until week 8.

Since its first marketing authorization nearly 40 years ago, super-high-potent and high-potent topical corticosteroids have been the most used and efficacious topical therapies available [17, 18], and are still the “cornerstone” of topical combination regimens for psoriasis [16]. Local and systemic AEs of topical corticosteroids include cutaneous atrophy, telangiectasia, burning/stinging, folliculitis, and suppression of the HPA axis [18]. Almost all TEAEs reported in this study were mild in severity, none of the AEs were considered definitely related to treatment by the investigators, and there were no SAEs or deaths. Although total systemic exposure to topical corticosteroids is one of the key concerns and this suppression is reported to be highest during the first week of treatment prior to healing of the skin lesions, this suppression is generally temporary and reversible [18]. In our study, as an indirect assessment of HPA axis suppression, mean plasma DHEAS levels in the GN-037 and CP arms did not show any difference from the V arm at weeks 2 and 4 and were comparable to baseline values in each arm at both timepoints. Furthermore, plasma CP was detectable only in 7.6% of the patients in the GN-037 and 2.8% of the patients in the CP arms at week 4 in this study, further supporting the minimum and limited systemic exposure, and therefore the lack of suppression of the HPA axis. Collectively, our findings demonstrated an excellent safety profile for the GN-037 cream.

Long-term management of MMPP with topical agents has been reported to be suboptimal; therefore, frequent relapses are inevitable [19]. Topical combination treatment options can provide better outcomes in reducing the occurrence of these relapses, and eventually, improving adherence to treatment in patients with MMPP.

The present study had certain limitations, such as the inclusion of patients with MMPP only or topical treatments not being used in combination with systemic therapies. Further studies are warranted to better determine the effectiveness and safety of GN-037 cream in patients with moderate-to-severe plaque psoriasis who are under systemic treatments.

## Conclusions

The results of this phase 2 double blind vehicle-controlled parallel group study indicate that GN-037 cream is an effective topical combination that achieves primary and secondary endpoints throughout the study. Safety data did not reveal any new safety concerns with the novel combination cream. Therefore, GN-037 is an effective and safe new treatment option for patients with MMPP who are not candidates for systemic treatments or who would benefit from adjuvant topical therapy in addition to systemic treatments.

## Data Availability

The datasets generated during and/or analyzed during the current study are available from the corresponding author on reasonable request.
